# Conformations of the Pyranoid Sugars. I. Classification of Conformers

**DOI:** 10.6028/jres.064A.017

**Published:** 1960-04-01

**Authors:** Horace S. Isbell, R. Stuart Tipson

## Abstract

An improved system is presented for indicating the principal conformations of pyranoid sugars and derivatives, by attaching two symbols to the systematic name. The *first* symbol shows the kind of pyranoid ring; for example, B = a boat, C = the chair, and S = a skew form. (The three boat and six skew rings are distinguished by subscript numerals referring to exoplanar ring-atoms.) The *second* symbol differentiates between two conformations that have the same type of ring, by describing as A or E the axial or equatorial character of the reference group at a selected ring-atom. If the anomeric group is not quasi, the *α* anomeric group is used as the reference group. For sugars and derivatives having a quasi anomeric group, and for relatives lacking an anomeric group, the A or E classification is based on the reference group at the lowest numbered, nonquasi, asymmetric ring-atom.

## 1. Introduction

Conformation, or molecular shape, is one of the most important properties of the sugars and related compounds. Consideration of the shape of a molecule and of how the various atoms and groups in it are oriented with respect to one another leads to a better understanding and interpretation of the reactions of the compound.

The term “*conformation*” was introduced into organic chemistry by Haworth [1]^1^, to describe the various shapes that certain molecules may assume. Conformations have been defined by Klyne [2] as “the different arrangements in space of the atoms in a single classical organic structure (configuration), the arrangement being produced by the rotation or twisting (but not breaking) of bonds.”

In contrast, the *configuration* of a compound is its unique spatial arrangement of the atoms in the compound, such that no other arrangement of these atoms is superimposable thereon to give complete correspondence, regardless of the conformation. Consequently, two compounds having *different* configurations may exist in the same general conformation; and the same compound (one configuration) may exist in different conformations.

If four atoms of the pyranoid ring are coplanar and the other two ring-atoms are *para* to each other, the latter atoms may lie on the same side (*cis*) or on opposite sides (*trans*) of the plane [3]. These two kinds of ring have been termed “boat” and “chair,” respectively.

Atoms and groups are attached to ring atoms by bonds which may differ as regards their angle to the plane of the ring (or a parallel plane). Such bonds [4, 5] are designated axial (*a*) [6], equatorial (*e*), and quasi (*q*) [7]. An axial bond lies nearly perpendicular to the plane; an equatorial bond, nearly in the plane; and a quasi bond, at about 55° to the plane. Quasi bonds occur in pairs and are equally inclined to the plane of the ring; they are not present in the boat and chair forms.

The projecting bonds of the ring atoms at the ends of the boat forms are not truly axial or equatorial; they have been called [8] “flagpole” (*fp*) and “bowsprit” (*bs*), respectively. (The terms *endo* and *exo* describe them more precisely, but, for the classification presented here, they will be regarded as being axial and equatorial, respectively.)

Although the importance of pyranoid conformations has been recognized for many years, their nomenclature has been inadequate. For lack of unambiguous conventions, confusion has arisen in applying the symbols used.

Classification of conformations necessarily involves consideration of configuration.

## 2. Naming of Anomers

Formation of an anomeric ring-form from an open-chain sugar creates a new asymmetric center at the reducing carbon atom; the resulting isomers (anomers) are designated *α* and *β.* Anomers are named from the relationship of the configuration at the anomeric carbon atom to the configuration of a reference carbon atom of the chain of the acyclic monosaccharide. If the chain has less than five asymmetric carbon atoms, the reference atom is the highest-numbered, asymmetric carbon atom. If the acyclic chain has more than four asymmetric carbon atoms, the reference atom is the highest-numbered asymmetric carbon atom in the group of four asymmetric carbon atoms next to the functional group. The anomer having the *same* configurations at the anomeric carbon atom and the reference carbon atom is named *α;* the anomer having *opposite* configurations at the two atoms is named *β.* The *α*-l isomer is the mirror image of the *α*-d isomer, and the *β*-l isomer is the mirror image of the *β*-d isomer.

Enantiomorphic sugars are classified into two configurational series, respectively designated d and l, according to the configuration of the reference carbon atom. In the Fischer projection formula, the hydroxyl group on the anomeric carbon atom of the or anomers (d and l series) lies in the same direction as the hydroxyl group on their reference carbon atom, i.e., to the right in the d series and to the left in the l series.

Although the Fischer projection formulas indicate the configuration at each asymmetric carbon atom, they do *not* show the positions assumed by the atoms in space. Perspective pyranoid formulas, originated by Drew and Haworth [9], do, however, approximately depict the positions (in a planar conformation) of all the atoms in the molecule with respect to the plane of the ring. On closing the pyranoid^2^ ring, carbon atom 5 of the open-chain sugar is rotated about its bond to carbon atom 4, so as to bring the ring-forming hydroxyl group into position for ring closure. With an *aldopentose*, turning carbon atom 5 in this manner merely causes a shift in the positions of the attached hydrogen atoms. With an *aldohexose*, so turning carbon atom 5 (to close the ring) causes the 5-*C*-(hydroxymethyl) group to assume a definite position with respect to the plane of the ring. When the ring is viewed in the manner shown, the 5-*C*-(hydroxymethyl) group of the d-hexopyranose lies above the plane of the ring and that of the l-hexopyranose lies below it. For the *aldohexopyranoses*, the *α* anomeric group is *trans* to the hydroxymethyl group, and the *β* anomeric group is *cis.* For the *aldopentopyranoses*, the *α* anomeric group is *cis* to the hydroxyl group at carbon atom 4 and the *β* anomeric group is *trans.*

**Figure:**
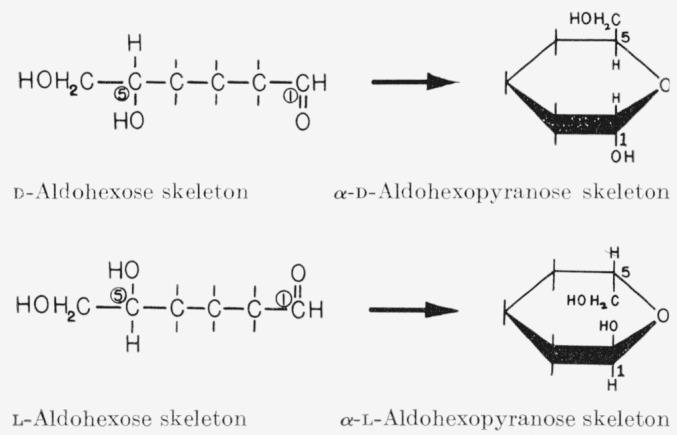


## 3. Early Work on the Conformations of the Pyranoid Sugars

After it had been shown [10] that the stable methyl glycosides of many aldopentoses, ketohexoses, and aldohexoses have the pyranoid ring, Sponsler and Dore [3] applied the concept of the d-glucopyranose [11] unit in interpretation of the X-ray diagram of cellulose. They realized that the conformations described for cyclohexane derivatives by Sachse [12] and by Mohr [13], namely, the chair and boat forms, could be *extended to pyranoid sugars.* They found that the chair conformations of the d-glucopyranose units satisfactorily explained the spacings in the X-ray diagram of cellulose, whereas the boat conformations did not.

In 1929, Haworth [1] further developed this theme. He pointed out that each pyranoid sugar is capable of existence in several strainless ring-forms of the Sachse–Mohr types. He discussed possible conformations, mentioned the form in which all the ring atoms are coplanar, and depicted four ring skeletons for the aldohexopyranoses, as in I–IV. He noted that

**Figure:**
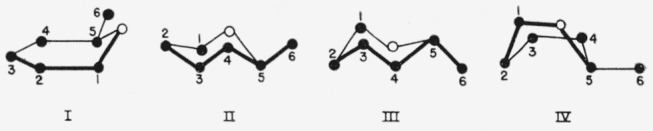

II and III “furnish two models for d-glucose and two for l-glucose,” and that there are six boat forms for d-glucose (and six for l-glucose), “making a total of sixteen possible arrangements for d- and l-glucose.”

At the time of the monumental work of Haworth and coworkers, the size, as well as the shape, of the ring in *the free sugars* was uncertain [14, 15]. In order to ascertain whether the pyranoid structures assigned (by methylation procedures) to “normal” methyl glycosides apply to the corresponding free sugars, a study of the mutarotation and oxidation reactions of aldoses was made by Isbell and coworkers [16, 17, 18]. The work had its origin in the observation of Isbell and Hudson [16] that aldoses can be oxidized by bromine to lactones *without cleavage and re-forming of the ring.* It was found that the free sugars, with few exceptions, give 1,5-lactones and have the pyranose structure.

Although considerable variation in the reactivity of the individual sugars with bromine was noted, the most striking difference was observed for the reactivity of the *α* and *β* anomers of the same sugar [19]. It was reasoned that the difference in reactivity arises from an important structural difference in the two, and that, if the pyranoid sugars have strainless ring-structures, such as Haworth had suggested earlier, the *α*and *β* hydroxyl groups must be inclined at different angles to the ring and therefore react at different rates. The authors pointed out that, if the sugars are divided into two groups, *α* and *β*, according to the position of the anomeric hydroxyl group with respect to the oxygen ring-atom, there is marked similarity in the rates of oxidation of the *α* sugars on the one hand, and of the *β* on the other [20, 21, 22]. This was the first recognition of the effect of conformation on reaction rates and the first attempt to classify sugar derivatives according to what are now known as axial and equatorial groups.

On the basis of similarity in properties, Isbell and coworkers sought to classify the sugars of like configuration and conformation. The mutarotation reactions and the rates of oxidation with bromine showed satisfactory correlations for crystalline l-arabinose (now named *β*-l-arabinose) with *α*-d-galactose, for *α*-d-xylose with *α*-d-glucose, and for *α*- and *β*-d-lyxose with *α*- and *β*-d-mannose, respectively. Classification of crystalline d-ribose was found to be less clear, and this sugar was first classified with *β*-d-allose and later with *α*-l-talose. Comparisons of the optical rotations of the sugars and glycosides revealed discrepancies in Hudson’s rotational difference (2A) that were ascribed to possible differences in conformation [21, 23].

When this study of the sugars was made, there was no known basis for predicting the stabilities of different conformations. The situation was changed by work on cyclohexane derivatives; this has been reviewed by Hassel [24], Orloff [25], and Mills [26]. The work of Hassel [4], Beckett and coworkers [5], and others led to recognition of certain factors that affect the stability of the various conformations of the cyclohexane ring. It was found that large groups attached to the *cyclohexane* ring tend to adopt (“prefer”) equatorial positions, and that the chair form of the ring is generally the most stable species.

Hassel and Ottar [27] extended these ideas to the *pyranoid* ring in 1947, and Reeves developed the matter more fully in a series of papers beginning in 1949. He clarified the subject and provided a firm foundation for much subsequent work [28 to 34].

## 4. Nomenclature of Conformations

**Figure 1:**
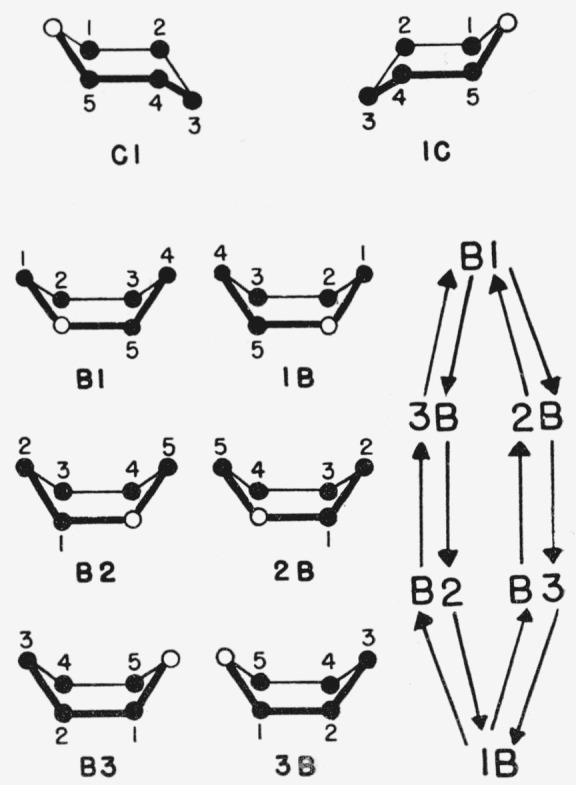
Reeves’ symbols [28] for the chair and boat forms of the pyranoid ring, and a diagram of possible pathways [34] for inter conversion of boat forms.

Reeves [28] depicted the two chair and six boat forms of the Sachse strainless-ring type (that had been discussed by Sponsler and Dore [3] and by Haworth [1]), and he assigned a symbol to each of these eight generalized, pyranoid rings as shown in figure 1. These rings consist of a chair ring, C1 (and its mirror image, 1C), and three boat rings (and their mirror images). The boat ring can be formed in three ways, according as the *cis* atoms displaced from the plane of the ring are carbon atoms **1** and 4, **2** and 5, or carbon atom **3** and the oxygen ring-atom; he designated these by the symbol B together with the numerals 1, 2, and 3, placed before or behind.

By his application of the symbols for the generalized structures (in fig. 1) to the d-glucopyranosides [28], with tabulation of bond angles, Reeves indicated that the symbols were to designate the rings of *the complete formulas containing the configurations.* In the same year, Reeves [29] showed the C1 and 1C forms of the d-hexopyranose ring with carbon atom 6 equatorial in the C1 and axial in the 1C form. He stated [29] that “the conformations involved are C1 in the d-galactopyranoside series, and, of course, the mirror image form 1C would apply to the l-galactopyranoside series.” Thus, the C1 and 1C symbols were used not only for the two different chair forms of any one pyranoid sugar, but also for enantiomorphs (having the same equatorial and axial distribution of groups).

Use of *different* symbols, for the *same* molecular shapes in mirror image, is self-consistent in this system of symbols, but is difficult for many students (and even workers in this field) to comprehend. In view of this situation, Isbell suggested [35] that the symbols C1 and C2 be used for the two different chair conformations of the pyranoses, regardless of their being in the d or l series. The important feature of the revised definition was that the same symbol applied, for both the d and the l configuration of a sugar, to those conformations having *the same axial and equatorial arrangement of groups.* In a later article, Isbell and coworkers [36] replaced the symbols C1 and C2 with C′1 and C′2 to avoid confusion with the C1 and 1C terms of Reeves. The authors pointed out that the axial and equatorial dispositions at each of the various ring-carbon atoms (in a strainless, pyranoid ring) have a fixed relationship to the conformation, and that the axial or equatorial disposition of the reference group attached to *any asymmetric carbon atom of the ring* could be used for description of a chair conformation. In the earlier classification, the C′1 and C′2 symbols were assigned to the chair forms according as *the reference group at carbon atom 5* was equatorial (C′1) or axial (C′2). As the aldopentopyranoses, ketohexopyranoses, and their deoxy derivatives lack a hydroxymethyl group at carbon atom 5, they could not be classified by this feature. They were, however, classified from a comparison of their physical properties with those of the structurally related aldohexopyranoses. This “classification” was empirical, and a change has seemed desirable.

Independently, Guthrie [37] and the present authors [38] have devised essentially the same solution to these problems in nomenclature. In our proposed nomenclature, the type of pyranoid ring is designated by C; B_1_ B_2_, B_3_; and S_1,3_, S_1,5_, S_2,0_, S_2,4_, S_3,5_, and S_4,0_. The symbols for the chair and boat forms are analogous to those of Reeves, except that symbol C includes his C1 and 1C; symbol B_1_ includes his B1 and 1B; symbol B_2_ includes his B2 and 2B; and symbol B_3_ includes his B3 and 3B. The symbols for the skew forms are new; the subscript numerals indicate the exoplanar ring-atoms.

**Figure 2:**
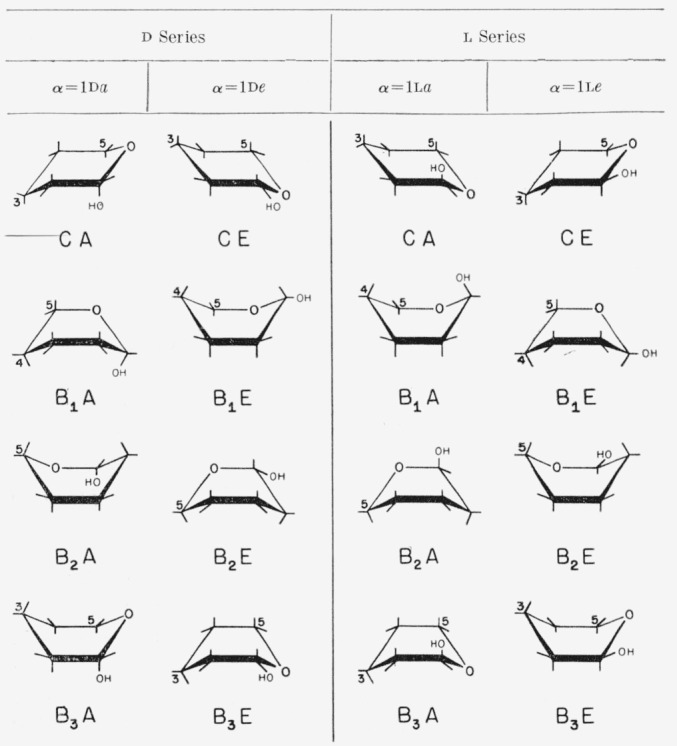
The chair and boat conformations of pyranoid sugars of the d and l series.

The anomeric carbon atom is common to all pyranoid sugars. If the disposition of the anomeric group is not quasi, it can be used for distinguishing between the two conformations of one ring type. The symbol describing the type of ring is combined with a second symbol, A or E, to indicate whether the *α* anomeric group is axial or equatorial.^3^ The two symbols are suffixed by a hyphen to the conventional name of the compound. For example, *α*-d-glucopyranose-CA indicates the chair conformation that has an axial anomeric hydroxyl group. The boat forms and those skew forms having a non-quasi anomeric group are named in like manner. The chair and boat conformations and the symbols proposed for them are given in figure 2.

All compounds classified earlier as C′1 or C′2 on the basis of the position of the reference group at carbon atom 5 are now classified as CA or CE, respectively. Compounds now designated as CA, CE, B_1_A, B_1_E, B_2_A, B_2_E, B_3_A, and B_3_E conformations have, in Reeves’ classification, C1, 1C, 1B, B1, B2, 2B, B3, and 3B rings, respectively, when in the d series; but, when in the l series, they have 1C, C1, B1, 1B, 2B, B2, 3B, and B3 rings, respectively.

The present system defines conformations of specific structures unambiguously, but the symbols A and E do not, of themselves, represent axial or equatorial dispositions. Capital letters A and E are used in the symbols, instead of lower-case letters, because *a* and *e* show the axial and equatorial dispositions of reference groups. Occasionally, it is advantageous to cite the actual dispositions of all of the reference groups; these may be given in numerical sequence without numbers. Thus, *α*-d-galactose-CA (V) is *α*-d-galactose-C(*aeeae*) and *α*-l-galactose-CE is *α*-l-galactose-C(*eaaea*). Formulas VI, VII, and VIII depict *β*(*a*)-l-arabinopyranose-CE, *α*-l-galactopyranose-CA, and *β*-d-arabinopyranose-CE; they may also be referred to as *β*-l-arabinopyranose-C*(aeea), α*-l-galactopyranose-C(*aeeae*), and *β*-d-arabinopyranose-C(*aeea*).

**Figure:**
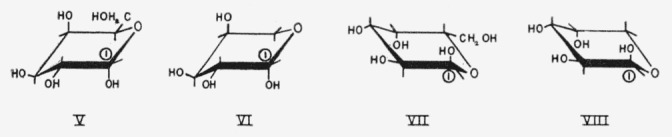


**Figure 3:**
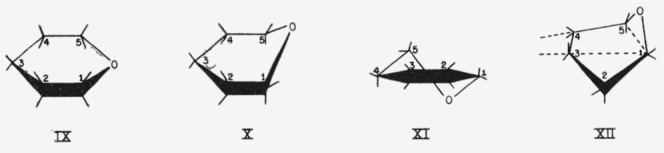
Examples of other possible pyranoid rings. IX, Planar; X, sofa; XI, half-chair *(trans-ortho);* XII, skewed (*trans-meta*); dotted lines indicate coplanar atoms.

In addition to the boat and chair rings already depicted (fig. 2), other rings, such as those shown in figure 3, are possible. In the *planar form* (IX), all of the ring-atoms are coplanar and all of the projecting bonds are quasi, although they have customarily been depicted as axial. In the *sofa forms* [39] (for example, X), all but *one* of the ring-atoms are coplanar; some of the projecting bonds of the sofa forms are quasi.

Pyranoid rings having *two* exoplanar atoms may be subdivided according as the relationship of these two atoms is cis or *trans*, and *ortho, meta*, or *para.* The *cis*- and *trans-para* forms (boats and chairs) have already been discussed. The *trans-ortho* forms (for example, XI) are the so-called *half-chair* forms [7]; the name was originally [40] applied to cyclohexene derivatives [7], but was extended [41] to pyranoid compounds. In these forms, four adjacent ring-atoms are coplanar. In the *trans-meta* (“stretched” [42] or “skewed” [34, 43]) forms, one ring-atom (of four coplanar ring-atoms) separates two ring-atoms that lie, respectively, above and below the plane, as in XII. In all the examples depicted in figure 3, some projecting bonds are quasi.

**Figure 4:**
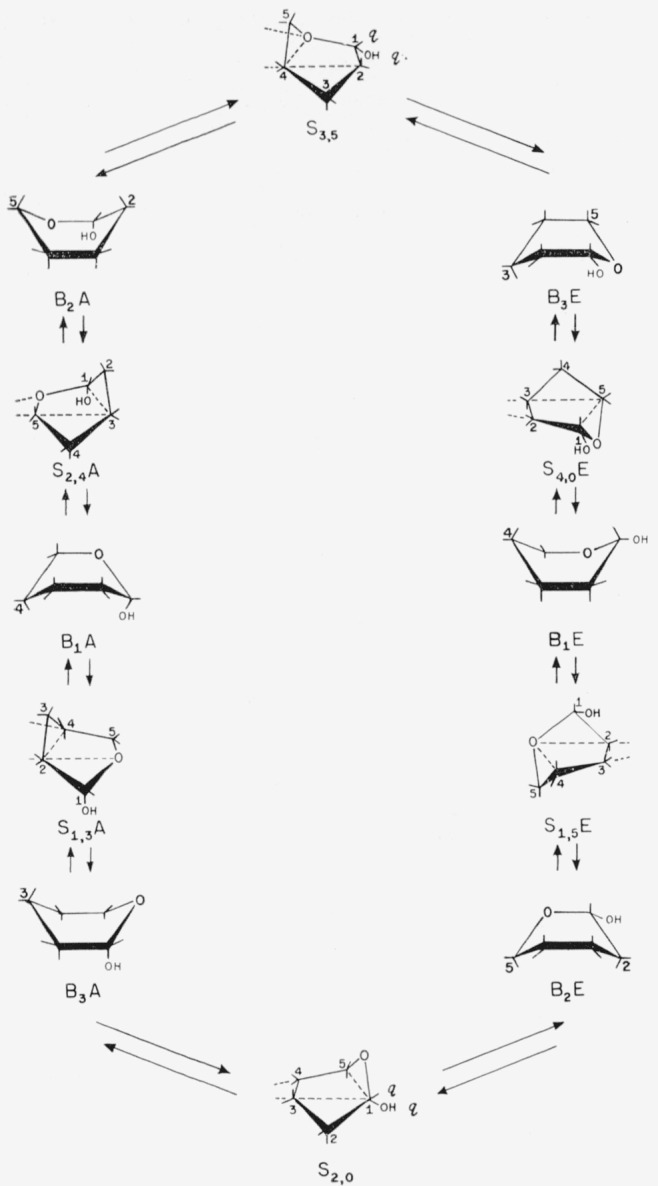
Pathways for the inter conversion of flexible forms of α-d sugars (in the boat-and-skew cycle proposed by Reeves [34]). B, Boat; q, quasi; S, skew; A, axial α anomeric group; E, equatorial α anomeric group.

According to Reeves [34, 43], the boat forms of the pyranoid ring, like the boat forms of cyclohexane discussed by Hazebroek and Oosterhoff [42], are less rigid than the chair forms. They are flexible and can be interconverted, without angular strain, through an infinite number of intermediate shapes. A skew form is midway between two boat forms.^4^ Figure 4 shows (with our formulas and symbols) the pathways proposed by Reeves for the interconversion of pyranoid boat- and skew-forms in a cycle. Each of the 12 forms shown has an enantiomorph (not depicted). It will be noted that the two skew forms having quasi bonds at carbon atom 1 (S_2,0_ and S_3,5_) are the turning points between the A and E conformations in the cycle. Although interconversion of the forms could take place without angular strain, other factors make the existence of a complete cycle improbable for many sugars and derivatives. Thus, energy barriers (arising from lion-bonded interactions, hydrogen bonding, resonance effects, or solvation) would tend to restrict the molecule to a definite form.

For conformations in which the projecting bonds at the anomeric carbon atom are quasi, assignment to an A or E series may be made on the basis of the reference group at the lowest numbered asymmetric carbon atom having axial and equatorial attachments. For example, *α*-d-idopyranose having the S_2,0_ conformation (XIII) would be called *α*-d-idopyranose-S_2,0_E_2_, where E_2_ refers to the equatorial (hydroxyl) reference group at carbon atom 2.

The conformations of the 1,5-anhydroalditols, 1,5-lactones, and all other pyranoid compounds lacking an anomeric carbon atom may similarly be classified. For example,

**Figure:**
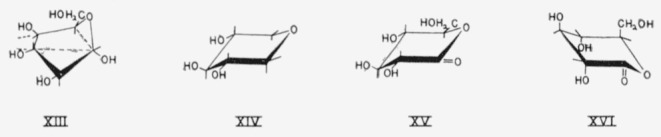

XIV is 1,5-anhydro-xylitol-CE_2_; and XY and XVI arc d-glucono-1,5-lactone-CE_2_ and d-glucono-1,5-lactone-CA_2_.

## Footnotes

1 Figures in brackets indicate the literature references at the end of this paper.

2 The terms “furanose” and “pyranose” were introduced by E. H. Goodyear and W. N. Haworth, J. Chem. Soc. 1927, 3136.

3 For added clarity, the disposition of the anomeric group can be further designated by insertion of (*a*) or (*e*) after the anomeric prefix. Ordinarily, this is not necessary.

4 R. D. Guthrie (private communication) points out that there are actually two skew forms between any two boat forms. We have depicted six of the twelve skew forms.
